# Dhurrin increases but does not mitigate oxidative stress in droughted *Sorghum bicolor*

**DOI:** 10.1007/s00425-022-03844-z

**Published:** 2022-02-28

**Authors:** M. N. Sohail, A. A. Quinn, C. K. Blomstedt, R. M. Gleadow

**Affiliations:** 1 School of Biological Sciences, Monash University, Clayton, VIC 3800 Australia; 2 Present Address: School of Natural Sciences, University of Tasmania, Private Bag 55, Hobart, TAS 7001 Australia

**Keywords:** Cyanogenic glucosides, Cyanide, Dhurrin, Drought, Phenolics, Reactive oxygen species

## Abstract

**Main conclusion:**

Droughted sorghum had higher concentrations of ROS in both wildtype and dhurrin-lacking mutants. Dhurrin increased in wildtype genotypes with drought. Dhurrin does not appear to mitigate oxidative stress in sorghum.

**Abstract:**

*Sorghum bicolor* is tolerant of high temperatures and prolonged droughts. During droughts, concentrations of dhurrin, a cyanogenic glucoside, increase posing a risk to livestock of hydrogen cyanide poisoning. Dhurrin can also be recycled without the release of hydrogen cyanide presenting the possibility that it may have functions other than defence. It has been hypothesised that dhurrin may be able to mitigate oxidative stress by scavenging reactive oxygen species (ROS) during biosynthesis and recycling. To test this, we compared the growth and chemical composition of *S. bicolor* in total cyanide deficient sorghum mutants (*tcd1*) with wild-type plants that were either well-watered or left unwatered for 2 weeks. Plants from the adult cyanide deficient class of mutant (*acdc1*) were also included. Foliar dhurrin increased in response to drought in all lines except *tcd1* and *acdc1*, but not in the roots or leaf sheaths. Foliar ROS concentration increased in drought-stressed plants in all genotypes. Phenolic concentrations were also measured but no differences were detected. The total amounts of dhurrin, ROS and phenolics on a whole plant basis were lower in droughted plants due to their smaller biomass, but there were no significant genotypic differences. Up until treatments began at the 3-leaf stage, *tcd1* mutants grew more slowly than the other genotypes but after that they had higher relative growth rates, even when droughted. The findings presented here do not support the hypothesis that the increase in dhurrin commonly seen in drought-stressed sorghum plays a role in reducing oxidative stress by scavenging ROS.

**Supplementary Information:**

The online version contains supplementary material available at 10.1007/s00425-022-03844-z.

## Introduction

Sorghum is the fifth most important grain crop in the world (FAOSTAT 2016) and is mainly used for grain and forage. Sorghum produces the cyanogenic glucoside dhurrin, which is non-toxic to the plant but upon tissue disruption, either by grazing or any physical process (e.g. freezing or conditioning for silage production), it is hydrolysed by dhurrinase, a β-glucosidase, releasing hydrogen cyanide (HCN) (Gleadow and Møller 2014). In nature this type of binary system, where two components are kept separate in their non-toxic forms, serves as a first line of chemical defence for plants against different herbivores and pathogens (Morant et al. 2008). Recent studies have proposed a nitrogen recycling pathway, which involves the breakdown of dhurrin without the release of any HCN (Nielsen et al. 2016; Pičmanová et al. 2015; Jenrich et al. 2007; Bjarnholt et al. 2018). This opens up the possibility for dhurrin to play additional roles besides defence, such as a source of nitrogen or the mitigation of stress.

The concentration of dhurrin has long been known to increase in response to drought stress, sometimes leading to death of livestock (Moaveni 2010; O'Donnell et al. 2013; Gleadow et al. 2016; Neilson et al. 2015). The total dhurrin mass is high even when the smaller size of the drought-stressed plants are taken into account (O'Donnell et al. 2013), suggesting that synthesis may be actively upregulated. The positive correlation between leaf dhurrin content and post-flowering drought tolerance in sorghum with the stay-green traits supports this view (Burke et al. 2013). Stay-green crops are ones with delayed senescence, allowing grains to continue to fill for longer, leading to increased yield. While dhurrin and stay-green are positively correlated, the relationship is not necessarily causal. A driver of stay-green is sustained nitrogen uptake from the soil during grain filling, delaying senescence (Borrell et al. 2001, 2014). Higher concentrations of dhurrin may, therefore, function as a nitrogen buffer in maturing plants, potentially delaying the need remobilise nitrogen from the leaves.

It is also possible that cyanogenic glucosides such as dhurrin may help plants to tolerate water deficits directly. Plants experiencing drought stress accumulate reactive oxygen species (ROS) in their leaves such as ^1^O_2_, H_2_O_2_, O˙^−^_2_, and OH˙ (Selmar and Kleinwächter 2013; Foyer and Noctor 2005; Choudhury et al. 2017; Kasson and Barry 2012). A number of mechanisms have been proposed to help plants to reduce the concentration of these harmful free radicals (Das and Roychoudhury 2014). One is to increase production of secondary metabolites, such as phenolics and cyanogenic glucosides (Akula and Ravishankar 2011; Jaleel et al. 2007; Neilson et al. 2015; Burke et al. 2013; Selmar and Kleinwächter 2013). This could be via the diversion of excess NADPH^+^, which accumulates as a result of stomatal closure and reduced CO_2_ flux. There could also be a more direct link. Sendker and Nahrstedt (2009), for example, found that the production of amides derived from cyanogenic glucosides increases with increasing H_2_O_2_ via the Radziszewski process. The proposed turnover pathway of dhurrin in sorghum also suggests amide formation (Nielsen et al. 2016; Pičmanová et al. 2015) hinting at the potential involvement of dhurrin in reducing RO- mediated stress. Thus, the process of amide formation from cyanogenic glucosides may function as a non-enzymatic scavenger mechanism in cyanogenic plants (Sendker and Nahrstedt 2009; Møller 2010; Schmidt et al. 2018).

To assess whether there was a causal link between dhurrin concentration and drought tolerance, we compared the growth and chemical composition of sorghum mutants that either lack dhurrin completely (*tcd1*, totally cyanide deficient) or have reduced capacity to accumulate dhurrin as they mature (*acdc1*, adult cyanide deficient class) with genotypes that have the usual dhurrin pathway. Dhurrin biosynthesis involves two cytochrome P450s (CYP79A1 and CYP71E1) (Koch et al. 1995; Sibbesen et al. 1995; Kahn et al. 1997; Bak et al. 1998) and a UDP-glycosyltransferase (UGT85B1) (Jones et al. 1999). The *tcd1* mutant has a mutation in the coding region of *CYP79A1*, which encodes the first and rate limiting enzyme involved in dhurrin biosynthesis (Blomstedt et al. 2012) and is acyanogenic throughout development. The *acdc1* mutant has mutation in the upstream regulatory region of *CYP79A1* (Blomstedt et al. 2012) and has normal levels of dhurrin in young plants, becoming increasingly acyanogenic as they mature. The five genotypes were grown in controlled environment greenhouses. Plants were divided into two groups when they reached the 3-leaf stage and either watered as normal, or left unwatered for 2 weeks. Plants were harvested and the concentration of ROS, dhurrin and total phenolics determined. Our hypothesis was that plants that make dhurrin would have lower concentration of ROS than mutant lines lacking dhurrin.

## Materials and methods

### Plant material, growing conditions and harvesting

Five genotypes of *Sorghum bicolor* (L.) Moench were included in the study: the publicly available line BTx623, two mutant lines [*adult cyanide deficient class 1* (*acdc1*) and *total cyanide deficient 1* (*tcd1*)], the breeding line that was mutated to produce the mutants (parent) and a sibling line of the mutated populations which has the same background but lacks the mutations (Blomstedt et al. 2012). The mutant lines, were developed at Monash University (Blomstedt et al. 2012). Both *acdc1* and *tcd1* mutants have a mutation in the regulatory and coding region of *CYP79A1* gene (Genebank accession numbers U32624), respectively. Plants were grown in a greenhouse under natural light conditions at Monash University during January and February 2018. The average day and night temperature was 28.15 ± 0.04 °C and 21.99 ± 0.05 °C, respectively, with an average light intensity of 242.57 ± 3.20 µmol m^−2^ s^−1^. All plants were grown in soil consisting of Debco seed raising mix (with trace elements and growth stimulants) and perlite (3:1 ratio). Plants (approx. 300) were germinated for each genotype in multiple thirty cell kwikpot^®^ trays (Garden City Plastics, Victoria). Forty-five plants per genotype, selected on the basis of uniform growth and developmental stage, were potted up into 75 mm pots (300 mL capacity) and watered every 2 days to saturation until the first baseline harvest and the start of the experimental treatment (details below).

#### First harvest (baseline)

Two weeks after germination, when plants had reached the 3-leaf stage, 14 plants of each genotype were harvested to provide baseline data prior to the application of the drought treatment. The harvested plants were divided into above ground (leaf blade and leaf sheath) and below ground (root) tissue, wrapped in foil and snap frozen in liquid nitrogen and stored at − 80 °C until freeze dried (DYNAVAC Freeze Drier Model# FD12). Due to their small size, two plants were pooled and considered as one sample and average weight was calculated (*N* = 7).

#### Second harvest (drought stress treatment)

After the baseline harvest, plants (3-leaf stage) were divided into two groups and either watered every 2 days with 60 mL water (well-watered treatment) or left unwatered (droughted), until plants reached the 5–6 leaf stage, 2 weeks after the start of the two watering regimes. Fifteen plants (*N* = 15) from each genotype and treatment were harvested and divided into: leaf blade, leaf sheath and roots following O'Donnell et al. (2013). Fresh weight was recorded. Plant material was then frozen in liquid nitrogen and freeze dried, as above. Freeze dried tissue was weighed and data used to calculate relative growth rate (RGR) (Eq. 1), and dry matter content (Eq. 2), as follows:

1 $${\text{RGR}}\;({\text{day}}^{ - 1} ) = \frac{{({\text{ln}}W_{2} - {\text{ ln}}W_{1} )}}{{(t_{2} - t_{1} )}}{, }$$

where, RGR is relative growth rate, *W*_1_ is total biomass at the first baseline harvest, *W*_2_ is total biomass at the second harvest and *t* is time.

2 $${\text{Dry}}\;{\text{matter}}\;{\text{content}}\;{ }({\text{\% }}) = \frac{{{\text{Dry }}\;{\text{weight}}}}{{{\text{Fresh }}\;{\text{weight}}}}{ } \times 100.$$

### Chemical analysis

Dhurrin concentration was determined by measuring the amount of hydrogen cyanide evolved from the tissue, with each milligram of HCN equivalent to 11.5 mg of dhurrin in the plant tissue based on the molecular weights of HCN (27 g mol^−1^) and dhurrin (311 g mol^−1^). To ensure all the dhurrin was converted to HCN, excess exogenous β-glucosidase (β-D-glucoside glucohydrolase, G4511, Sigma-Aldrich, Sydney, Australia) was added to finely ground freeze-dried tissue (9.5–10.5 mg). Evolved HCN was captured as NaCN in a 1 M NaOH solution and measured via a colorimetric assay (Woodrow et al. 2002). Absorbance was measured at 580 nm using a microplate reader spectrophotometer (FLUOstar Galaxy, BMG Labtech). The cyanide concentrations were determined by comparison to the NaCN standard curve included in each microtitre plate and converted to mg g^−1^ Dry Weight (DW). Assays were done in triplicate. Quantification of HCN using this method is equivalent to direct measurement of dhurrin by LCMS (Gleadow et al. 2012). The level of free HCN in plant tissues was assumed to be negligible (Gleadow et al. 1998). Concentrations of HCN below 10 ppm are considered to be ineffective in herbivore defence (Gleadow and Woodrow 2002a; Gleadow et al. 2003).

Phenolic concentrations were determined following Burns et al. (2002) modified as following: 10 mg of finely ground dried tissue, extracted in 1 mL of cold 70% v/v acetone with homogenisation in a MM 300 MixerMill (Retsch, Haan, Germany) at 30 rps for 1 min. The supernatant was collected after centrifugation (15,000*g*) at 4 °C for 20 min and 20 μL of each sample placed in 96-well microplate wells, followed by addition of 20 μL distilled H_2_O. Folin–Ciocalteu reagent (20 μL: Sigma F-9252; diluted 1:2 with deionized H_2_O) was added to each well, followed by 200 µL of 2% Na_2_CO_3_ in 0.1 M NaOH. Samples were incubated for 120 min at room temperature and the absorbance read at 740 nm using a Fluostar Galaxy plate reader (BMG Labtech, Australia). Gallic acid (Sigma-Aldrich) was used as the standard (range 0–250 µg mL^−1^) and the results expressed as gallic acid equivalents per milligram (dry weight) of tissue. All assays were done in triplicate.

Reactive oxygen species (ROS) were measured based on oxidation of non-fluorescent 2′,7′-dichlorodihydrofluoresein (DCFH_2_) (Sigma-Aldrich) to the fluorescent form 2′,7′,-dichlorofluoreocein (DCF) (Sigma-Aldrich) (Keston and Brandt 1965) modified as follows: stocks for 0.25 mM H_2_DCF-DA and 50 µM DCF were made up in absolute ethanol and stored in the dark at − 20 °C. Freeze dried tissue was ground to a fine powder using a Mixer Mill 301 (Retsch) at 28 oscillations per seconds for 30 s. Samples were weighed to 15 mg (± 5%) in 1.7 mL microcentrifuge tubes and all subsequent steps were performed in the dark at 4 °C. For extraction, 1 mL of cold 40 mM TRIS–HCl buffer (pH 7.0) was added to the sample, before vortexing for 30 s and centrifugation for 15 min at maximum speed in a pre-cooled rotor (4 °C). Supernatant was carefully removed and collected in a separate 10 mL centrifuge tube. The extraction was repeated for each sample as above, with the supernatants combined in the same centrifuge tube along with an additional 1 mL TRIS buffer before mixing. After extraction, 200 μL of each sample was aliquoted in triplicate onto a 96-well black microtiter plate (Greiner Bio-One), followed by 20 μL of 0.25 mM H_2_DCF-DA. Plates were sealed with laboratory film and covered with aluminium foil before incubation at 37 °C for 90 min to allow oxidation of H_2_DCF to DCF by intercellular ROS**.** DCF standards (ranging from 0 to 2500 nM) were prepared in TRIS buffer and 220 μL of each was added to the plate in triplicate. Fluorescence was measured using a fluorescence spectrophotometer (FLUOstar Galaxy, BMG Labtech, Australia) with excitation and emission wavelengths set at 485 nm and 525 nm, respectively. A standard curve was used to convert concentration of DCF to ng mg^−1^ DW.

### Statistical analysis

Data was analysed using Multivariate ANOVA and Pearson’s correlation coefficient, in IBM SPSS Statistics V25 (Corp 2017). Means were compared post hoc using Tukey tests.

## Results

### Growth of watered plants varies with age and genotype but droughted plants were similar

Plants were watered regularly until the 3-leaf stage, when they were divided into two groups: watered and drought-stressed. At the 3-leaf stage BTx623 had the highest total plant biomass 124 ± 4 mg, significantly greater than the other four genotypes (Table 1). The *tcd1* mutants, which lack dhurrin, had the lowest total biomass (61 ± 5 mg), significantly lower than the sibling line with the same genetic background (Table 1). This was primarily due to the difference in shoot mass, which was significantly higher in *acdc1*, the sibling line and BTx623 compared with *tcd1* (Table 1).

**Table 1:** Plant biomass partitioning growth of five different genotypes of *Sorghum bicolor* (parent, BTx623, *acdc1*, *tcd1* and sibling) from the first (3-leaf stage) harvest

Parameter	Genotype				
	Parent	BTx623	*acdc1*	*tcd1*	Sibling
Root dry biomass (mg)	18 ± 1^c^	34 ± 1^a^	23 ± 1^bc^	21 ± 3^bc^	24 ± 2^b^
Shoot dry biomass (mg)	48 ± 2^bc^	90 ± 4^a^	55 ± 2^b^	38 ± 3^c^	56 ± 2^b^
Whole plant dry biomass (mg)	66 ± 3^bc^	124 ± 4^a^	74 ± 5^bc^	61 ± 5^c^	80 ± 3^b^
Root:shoot ratio	0.37 ± 0.01^b^	0.38 ± 0.02^b^	0.41 ± 0.03^ab^	0.53 ± 0.06^a^	0.43 ± 0.03^ab^

Fourteen plants from each genotype were harvested but due to their small size, two plants were pooled and considered as one sample and average weights for each tissue type were calculated (*N* = 7). Means with the same letter are not significantly different at *p* < 0.05 using Tukey’s pairwise comparisons

**Fig. 1:**
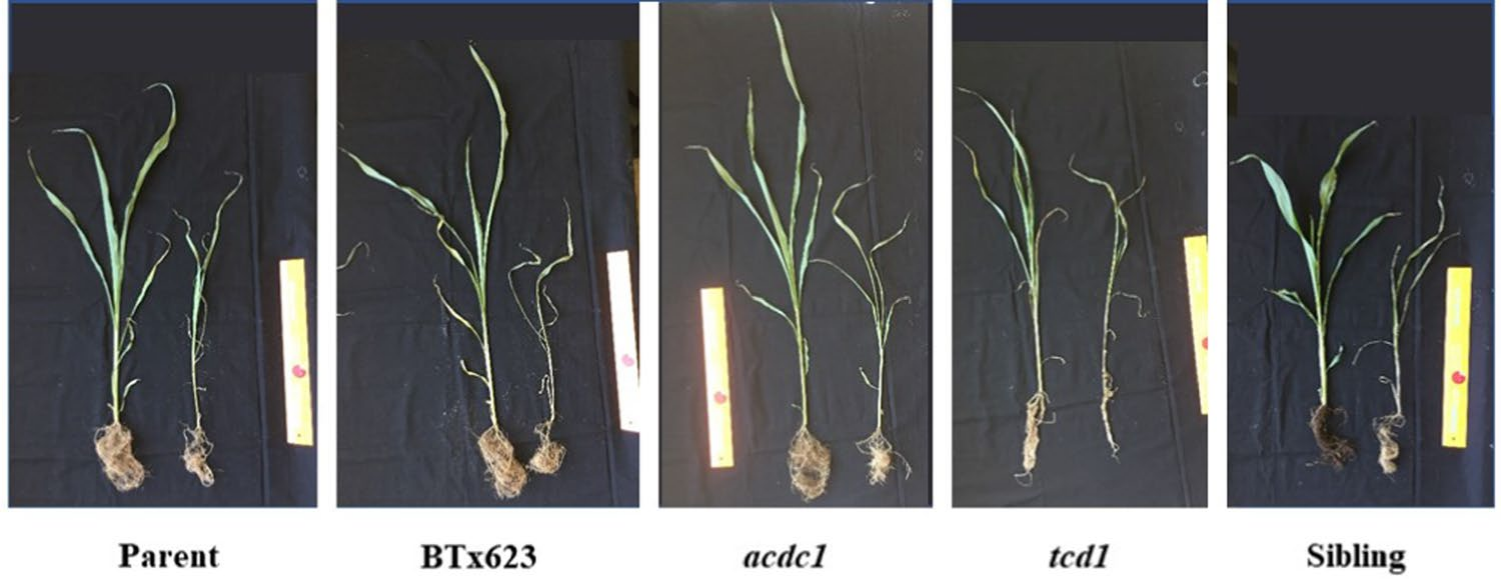
Growth of wildtype and EMS mutants of *Sorghum bicolor* grown under well-watered conditions or left unwatered for 15 days after plants reached the 3-leaf stage (drought). Plants on the left in each panel are the well-watered control treatment. From left to right parental line of EMS mutants; BTx623; *acdc1*, adult cyanide deficient class; *tcd1*, totally cyanide deficient; sibling line with the same background as the mutants, but without mutations in the dhurrin pathway. Scale = 30 cm ruler

**Fig. 2:**
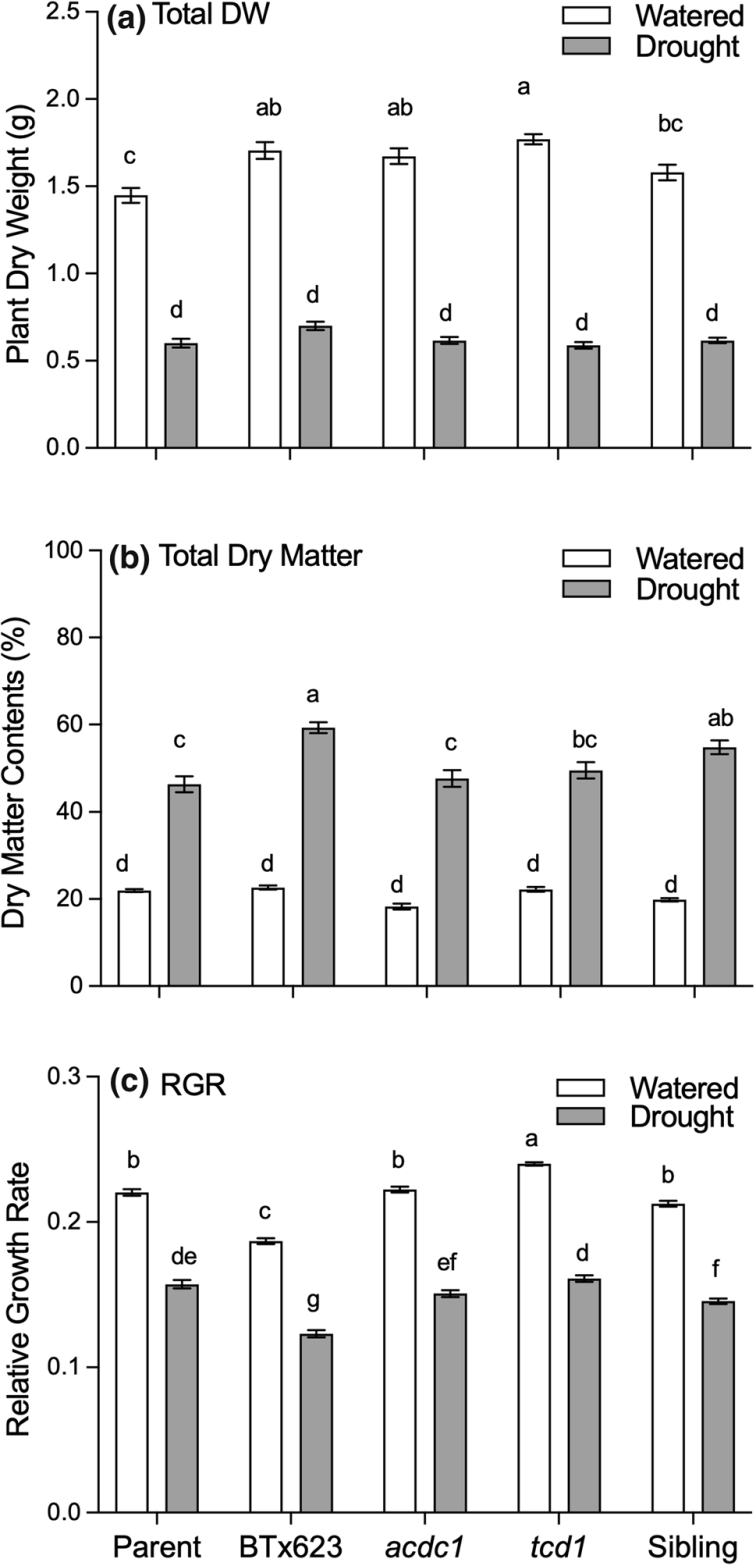
**a** Total biomass (dry weight), **b** dry matter content and **c** relative growth rate of five *Sorghum bicolor* genotypes (parent, BTx623, *acdc1*, *tcd1* and sibling) grown under well-watered conditions or drought conditions. *tcd1* plants lack dhurrin completely. Plants in the sibling line have the same genetic background as the mutants but without the mutation. Plants were harvested at the 5–6 leaf stage. Values are the mean of 15 replicates ± SE. Columns with the same letter are not significantly different from other columns on the same graph (*p* < 0.05)

Plants were allowed to grow for 15 days under the two treatments and either watered normally or left unwatered. Drought stressed plants were smaller and, on average, one leaf stage behind (5-leaf stage) than the well-watered plants (6-leaf stage) (Figs. 1, 2; Supplementary Fig. S1). RGR decreased by about one third in droughted plants compared to the plants grown under well-watered conditions (Fig. 2c). In contrast to the results at the 3-leaf stage, the total biomass of the *tcd1* plants under well-watered conditions was higher than the parental and sibling lines, not smaller (Fig. 2a). The *tcd1* genotype also had the highest relative growth rate under both well-watered (0.240 ± 0.001) and droughted (0.161 ± 0.002) conditions (Fig. 2c). Total biomass was similar for plants of all genotypes that were left unwatered (Fig. 2a). This was also the case for the root:shoot ratio (Supplementary Fig. S2).

Plant dry matter content is a measure of both the water status of the plant and the suitability of the forage for grazing. Dry matter content was more than double in droughted plants of all genotypes compared to the water controls (Fig. 2b, Supplementary Fig. S3). In droughted plants, BTx623 and the sibling line had the highest levels of dry matter content, irrespective of tissue type. Watered plants were not significantly different in dry matter content (Fig. 2b, Supplementary Fig. S3).

### HCNp is higher in leaves of wildtype plants that had been droughted, but not in other tissues

**Fig. 3:**
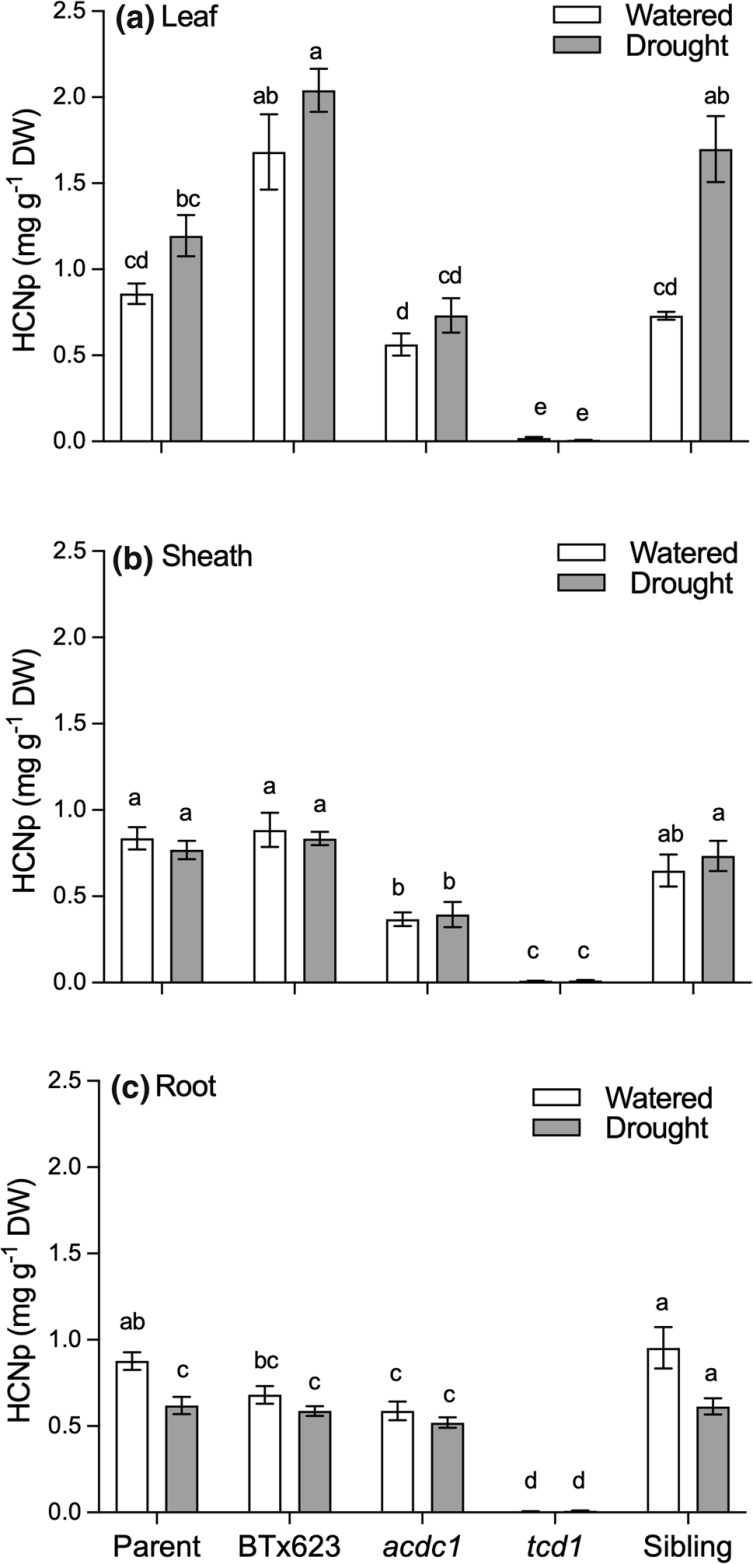
Concentration of hydrogen cyanide (HCNp) of **a** leaves, **b** sheaths and **c** roots of five *Sorghum bicolor* genotypes at the 5–6 leaf stage (parent line, BTx623, *acdc1*, *tcd1* and sibling) grown under well-watered or drought conditions. *tcd1* plants lack dhurrin completely. Plants in the sibling line have the same genetic background as the mutants but without the mutation. Values are the mean of 15 replicates ± SE. Columns with the same letter are not significantly different from each other (*p* < 0.05)

Dhurrin concentration was measured as HCNp, the total amount of hydrogen cyanide evolved from the leaves (leaf blades), leaf sheaths, and roots of five different sorghum genotypes (Fig. 3). HCNp varied with genotype and tissue type. The highest HCNp was observed in the leaves of BTx623, compared to all other lines. All tissues of *tcd1* plants, which lack a functional CYP79A1 enzyme, were acyanogenic, with only trace levels of HCN detected (Fig. 3). HCNp was almost 50% lower in leaves and sheath of *acdc1* plants, but roots had similar concentrations to the wildtype lines. Foliar HCNp was higher in droughted plants of all genotypes that were cyanogenic, including the *acdc1* mutant line, although the difference was only significant in the sibling line. In the sheath tissue, there was no significant increase in HCNp with drought treatment in any genotype (Fig. 3b). However, in the root tissue there was a significant reduction in HCNp in the parent and sibling lines exposed to drought (Fig. 3c).

**Fig. 4:**
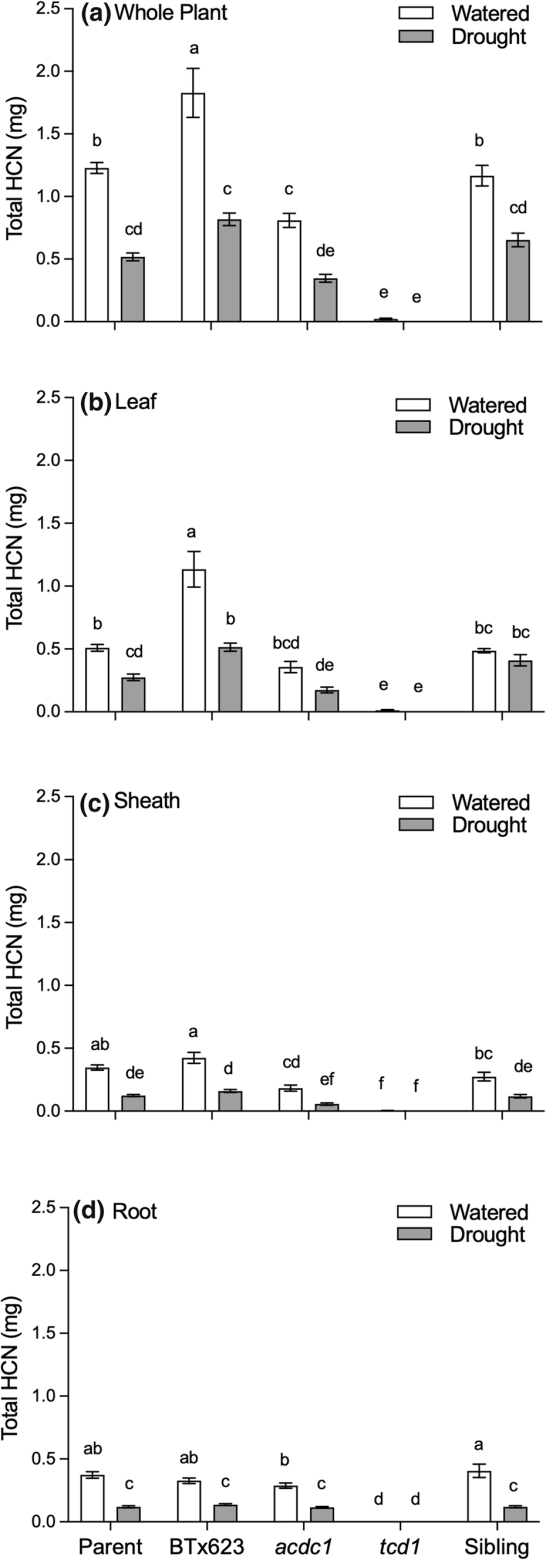
Total amount of hydrogen cyanide expressed on a per plant (whole plant) or per tissue basis for five different *Sorghum bicolor* genotypes (parental line, BTx623, *acdc1*, *tcd1* and siblings) grown under well-watered or drought conditions; *tcd1* plants lack dhurrin completely. Plants in the sibling line have the same genetic background as the mutants but without the mutation. Values were calculated by multiplying the concentration of each metabolite by the biomass of each tissue. Values are mean of 15 replicates ± SE. Bars with the same letter are not significantly different from each other (*p* < 0.05)

To account for differences in plant size, the total amount of hydrogen cyanide in each plant was calculated by multiplying HCNp for each tissue by the mass. On a whole plant basis, the total amount of hydrogen cyanide was significantly lower in plants that had been droughted (*P* < 0.05), except *tcd1*, which is acyanogenic (Fig. 4a). This trend was the same in all tissues, except for the leaves of *acdc1* and sibling lines, where the total cyanide was not significantly different under well-watered and drought conditions (Fig. 4b–d).

### Concentration of ROS increases in leaves of droughted plants but not in the sheaths or roots irrespective of genotype

**Fig. 5:**
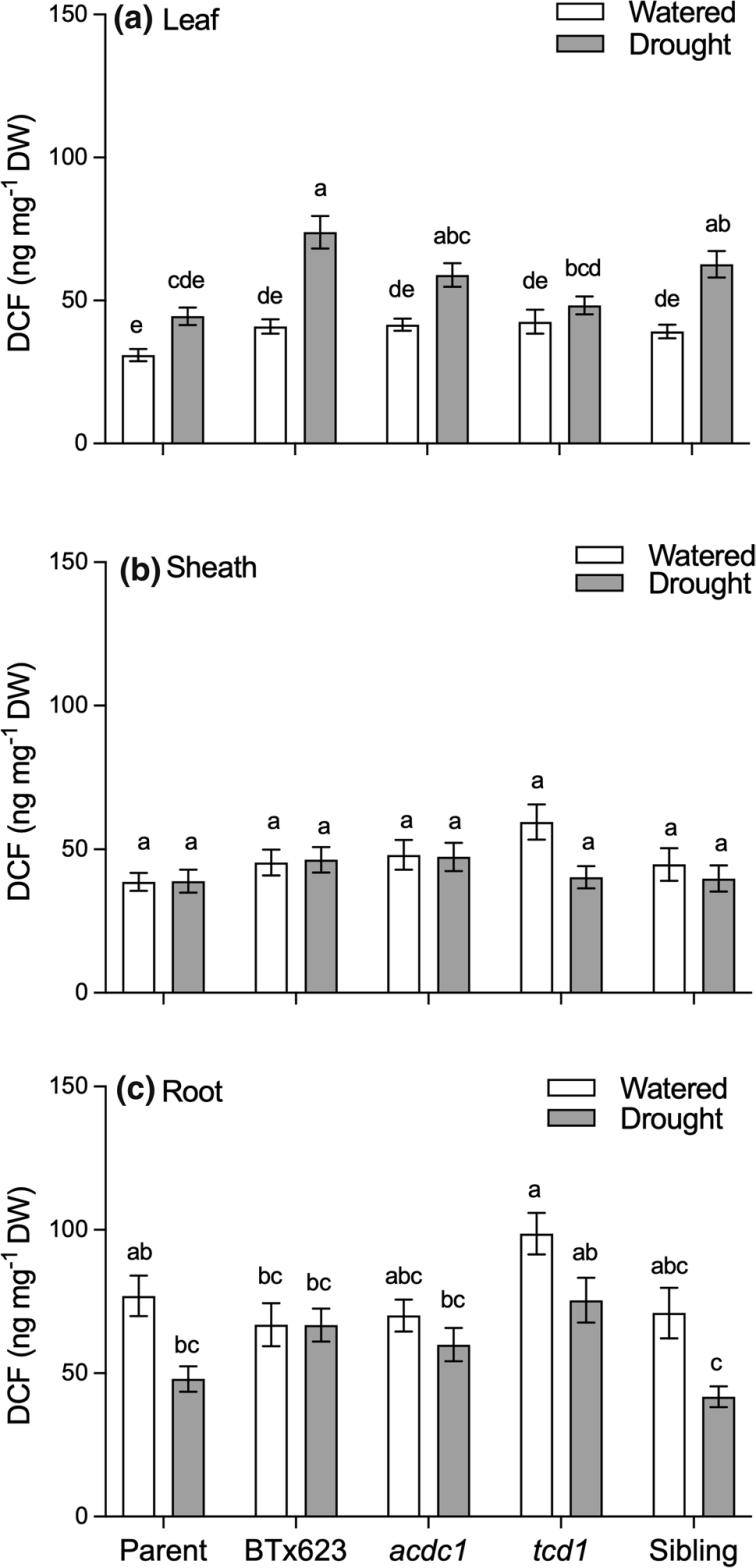
Concentration of ROS (measured as DCF equivalents) of **a** leaves, **b** sheaths and **c** roots of five *Sorghum bicolor* genotypes at the 5–6 leaf stage (parent line, BTx623, *acdc1*, *tcd1* and sibling) grown under well-watered or drought conditions. *tcd1* plants lack dhurrin completely. Plants in the sibling line have the same genetic background as the mutants but without the mutation. Values are the mean of 15 replicates ± SE. Columns with the same letter are not significantly different from each other (*p* < 0.05)

The concentration of ROS was similar among genotypes in well-watered plants, for leaves, sheaths and roots (Fig. 5). There were significant increases in foliar ROS in droughted compared to well-watered plants of Btx623, *acdc1* and the sibling genotypes, but not the parent line or *tcd1* where the increase was not significant (Fig. 5a). By contrast, ROS concentration in the sheath tissue was unchanged in all genotypes from the drought treatment (Fig. 5b), and somewhat lower in the roots of the parent and sibling lines (Fig. 5c).

**Fig. 6:**
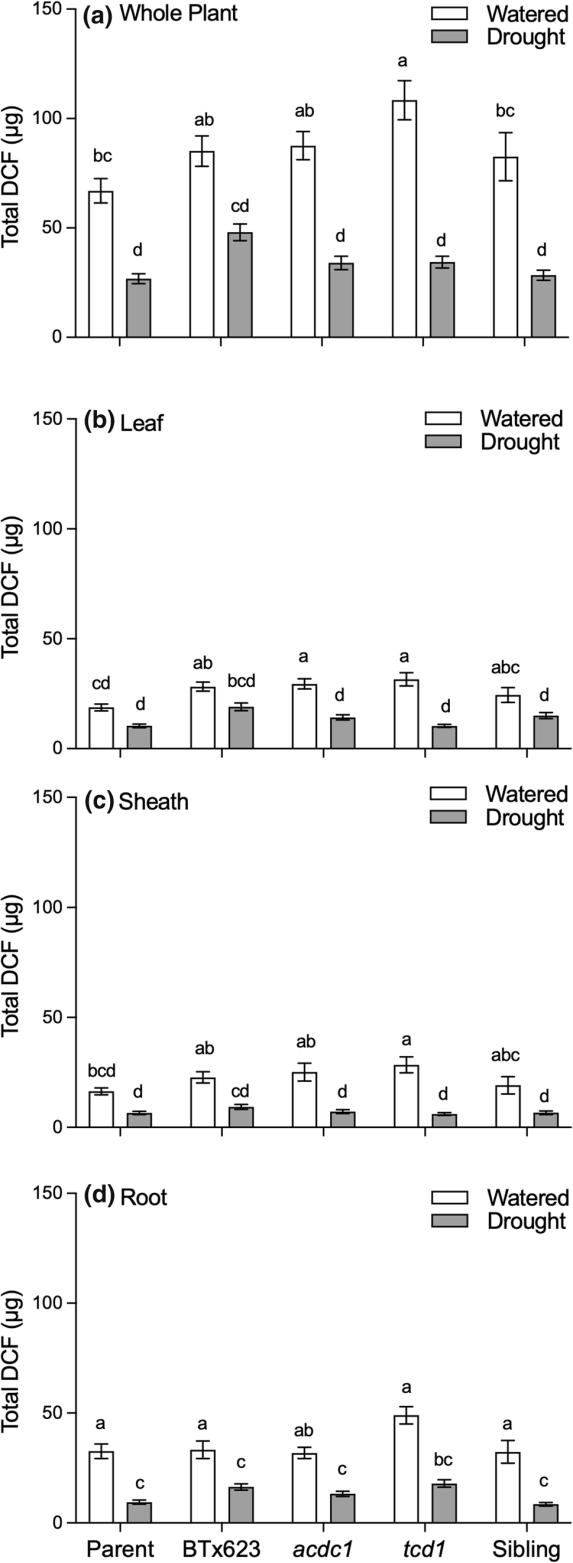
Total amount of reactive oxygen species (ROS) expressed on a per plant (**a**, whole plant) or per organ basis (**b**–**d**) for five different *Sorghum bicolor* genotypes (parental line, BTx623, *acdc1*, *tcd1* and siblings) grown under well-watered or drought conditions; *tcd1* plants lack dhurrin completely. Plants in the sibling line have the same genetic background as the mutants but without the mutation. Conversion of dichlorofluorescein diacetate (DCF-DA) to dichlorofluorescein (DCF) by oxidation enables quantification of ROS using fluorescence spectrophotometry. Values were calculated by multiplying the concentration of each metabolite by the biomass of each tissue. Values are mean of 15 replicates ± SE. Bars with the same letter are not significantly different from each other (*p* < 0.05)

ROS levels were calculated on a whole plant basis by multiplying concentration by biomass of the relevant plant part (Fig. 6). Whole plant ROS was significantly lower in droughted plants, due to their smaller biomass (Fig. 6a). There were some small differences between genotypes under well-watered conditions on a whole plant basis, but this was not readily attributable to difference in any particular tissue. Under drought conditions there were no significant differences in total ROS in any tissue type for any genotype.

### Total phenolics were similar among genotypes and did not vary between treatments

**Fig. 7:**
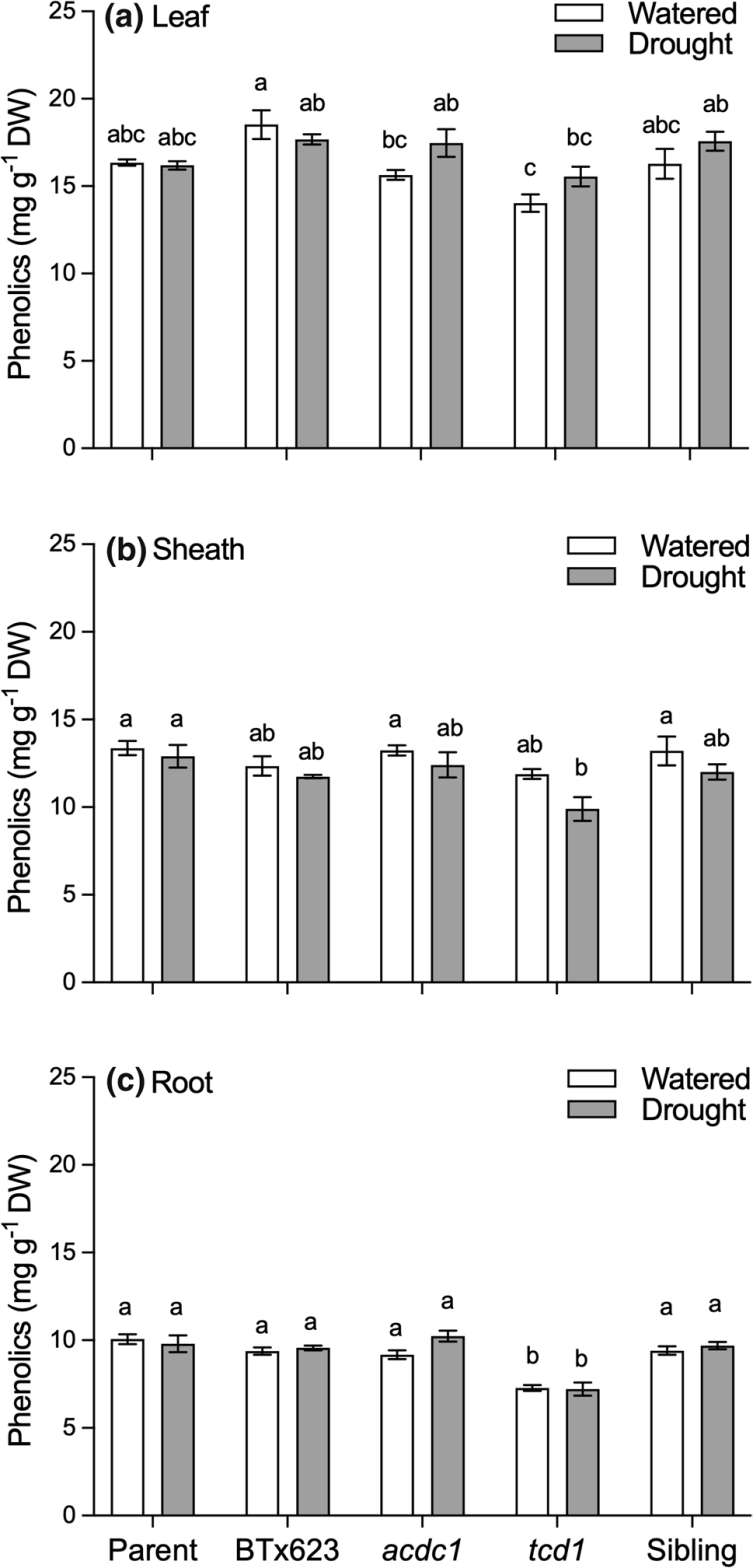
Concentration of total phenolics of **a** leaves, **b** sheaths and **c** roots of five *Sorghum bicolor* genotypes at the 5–6 leaf stage (parent line, BTx623, *acdc1*, *tcd1* and sibling) grown under well-watered or drought conditions. *tcd1* plants lack dhurrin completely. Plants in the sibling line have the same genetic background as the mutants but without the mutation. Values are the mean of 15 replicates ± SE. Columns with the same letter are not significantly different from each other (*p* < 0.05)

**Fig. 8:**
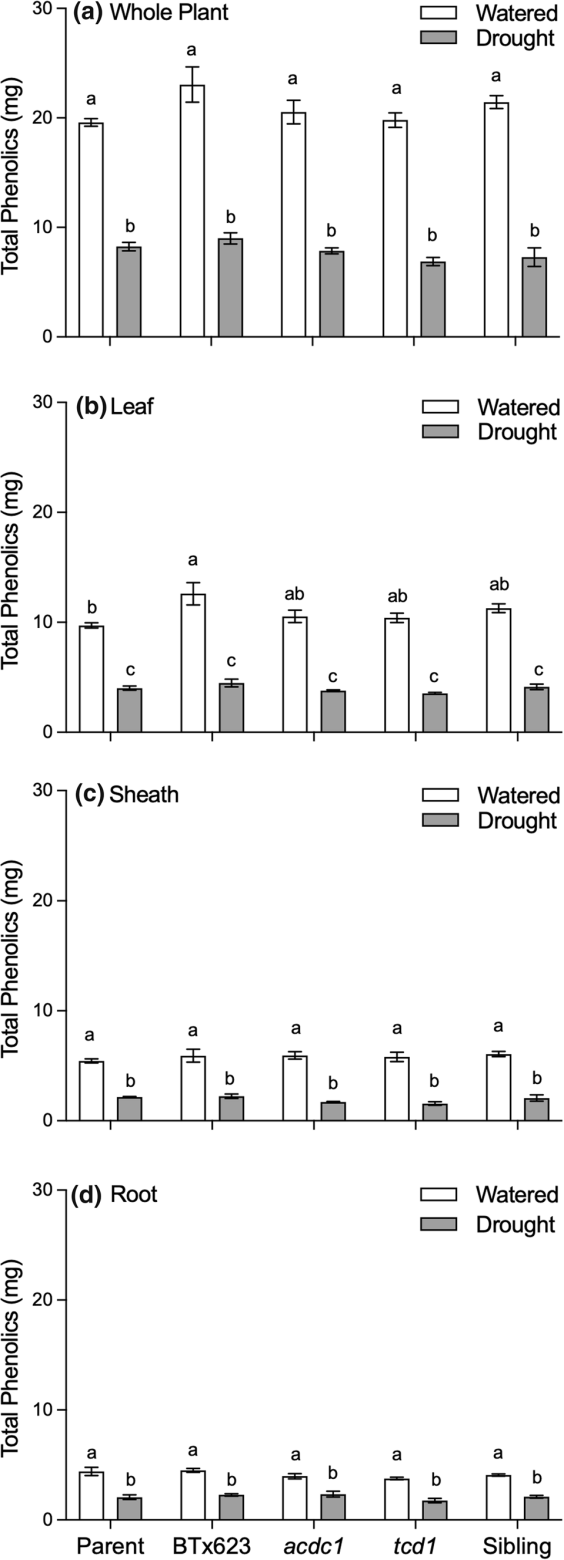
Total amount of phenolics expressed on a per plant (whole plant) or per organ basis for five different *Sorghum bicolor* genotypes (parental line, BTx623, *acdc1*, *tcd1* and siblings) grown under well-watered or drought conditions; *tcd1* plants lack dhurrin completely. Plants in the sibling line have the same genetic background as the mutants but without the mutation. Values were calculated by multiplying the concentration of each metabolite by the biomass of each tissue. Values are mean of 15 replicates ± SE. Bars with the same letter are not significantly different from each other (*p* < 0.05)

Under well-watered conditions, foliar phenolic concentration was significantly lower in the two mutants, *acdc1* and *tcd1,* which have reduced or no HCN, respectively, than BTx623 (Fig. 7a). However, there was no significant difference across genotypes in phenolic concentration in the leaves of the droughted plants. In the sheath tissue there was no significant difference among genotypes or treatment, except for the droughted *tcd1* plants, which had a significantly lower concentration of phenolics than the parent line (Fig. 7b). Root phenolic concentration was also significantly lower in *tcd1* in both watered and droughted plants (Fig. 7c). We calculated the amount of phenolics on a whole plant basis, and as with the other chemicals measured, the total amount of phenolics in each plant was less in the droughted plants, due to their smaller size. There were no differences among genotypes on a whole plant basis, or for phenolic content of each of the three tissue types under the two watering treatments (Fig. 8).

## Discussion

In this study, we used sorghum mutants which completely lacked dhurrin (*tcd1*) or had lower levels (*acdc1*) to test the hypothesis that higher concentrations of cyanogenic glucosides help mitigate the increase in ROS often observed in drought-stressed plants. To do this we compared ROS, dhurrin and total phenolic concentrations in different plant tissues from five different genotypes of *S. bicolor* that were either watered normally or left unwatered for 15 days (drought treatment). Physiological and morphological parameters demonstrate that the unwatered plants were severely drought affected. All genotypes in the drought treatment were stunted and at least one leaf stage behind the plants in the well-watered treatment group (Figs. 1, 2). Dry matter content (the inverse of water content) also increased in all genotypes (Fig. 2b).

### Dhurrin is associated with faster growth in very young plants

The *tcd1* plants had the lowest whole plant biomass at the 3-leaf stage but by the 5-leaf stage they had the greatest RGR under both well-watered and drought conditions, resulting in an overall similar biomass. Delayed growth was not observed in the *acdc1* mutants that contain similar concentrations of dhurrin to the wildtype when young or in the sibling line which has the same background, but lacks the mutation. This reduced growth of *tcd1* at the earliest stages of plant development is consistent with previous results investigating this particular genotype under differing nitrogen regimes (Blomstedt et al. 2018; Sohail et al. 2020). Our experiment did not look at whether these mutants were more vulnerable to herbivores. Mature seeds do not contain dhurrin; rather, it is synthesised rapidly on imbibition with concentrations as high as 35% on a dry mass basis in etiolated coleoptiles (Halkier and Møller 1989). Young emerging tissues are vulnerable to being eaten and presumably the very high levels of dhurrin are playing a protective role (Gleadow and Woodrow 2002a). However, the repeated observation that very young *tcd1* plants are smaller points to alternative roles, e.g. in smoothing the supply of nitrogen. Other cyanogenic species such as *Manihot esculenta* that have been engineered to become acyanogenic also show slower growth in early development (Jørgensen et al. 2005).

### Mutants deficient in cyanide were no more adversely affected by drought than wildtype plants

HCNp was higher in the leaves of all the wildtype plants that were left unwatered (Fig. 3a). Similar results have been observed before in drought-stressed *S. bicolor* and is a common response in cyanogenic plants from a wide range of families (Gleadow and Woodrow 2002b; Brown et al. 2016; Gleadow et al. 2016). The amount of dhurrin produced by sorghum in response to drought also depends on the intensity and duration of the stress. O'Donnell et al. (2013) found that plants under 10% PEG induced osmotic stress showed the same HCNp as the control (0%) treatment, but plants under 20% PEG treatment showed significantly higher HCNp as compare to both control and 10% PEG. Emendack et al. (2018) found a reduction in dhurrin in response to brief water stress but prolonged stress increased the dhurrin concentration. The *tcd1* mutant plants in the present study remained totally deficient in cyanide regardless of the treatment. Interestingly, the *acdc1* mutants, which retain the capacity to synthesise dhurrin, did not show any significant increase in HCNp with drought. The reason for this is unclear, but it could be that the putative mutation in the regulatory region of the *acdc1* mutant described by Rosati et al. (2019) makes it insensitive to drought stress.

### Dhurrin and phenolics do not appear to moderate ROS in the leaves of drought-stressed plants

We predicted that plants that were able to synthesise dhurrin would be more drought tolerant than those that lacked dhurrin due to their purported ability to scavenge ROS. In our experiment, foliar ROS concentrations increased in plants that were left unwatered for 2 weeks, irrespective of whether the plants produce dhurrin or not (Fig. 5). Moreover, while there were no significant differences in ROS concentrations in the sheaths and roots of droughted plants, there was a tendency towards lower concentrations in *tcd1* plants, rather than the higher concentrations we had predicted. To assess whether there was a correlation between the actual amounts of dhurrin and ROS we multiplied the concentration by the mass for each tissue type. Total amounts of ROS and dhurrin per plant basis were lower in all drought-stressed plants due to their smaller biomass. There were no significant differences among genotypes on a whole plant basis either.

At any given time, the ROS concentration in cells depends on the difference in the rate of its production and scavenging (Mittler et al. 2004; Das and Roychoudhury 2014). The most common plant response to drought stress is the closure of stomata to minimize water loss, which also results in reduced CO_2_ intake and leads to an imbalance between light harvesting and carbon fixation. The associated accumulation of NADP^+^ reducing power can lead to increases in ROS generation, which is potentially damaging to organelles (Das and Roychoudhury 2014). It has been hypothesised that this excess energy can be dissipated through passive channelling of NADP^+^ towards the synthesis of specialised metabolites (Selmar and Kleinwächter, 2013), including but not limited to cyanogenic glucosides (Neilson et al. 2013). Sendker and Nahrstedt (2009) suggested that the cyanogenic glucosides prunasin may be converted to its primary amide glucoside, prunasinamide in drying and decaying leaves via a reaction with H_2_O_2_ known as the Radziszewski process. If so, this could explain how recycling of cyanogenic glucosides could mitigate oxidative stress. Primary amide glucoside derivatives have also been identified in sorghum (Montini et al. 2020; Nielsen et al. 2016; Pičmanová et al. 2015), as well as cassava and almond (Pičmanová et al. 2015). Our results do not support this mechanism as being important in *S. bicolor* under the type of drought imposed in this study, it is possible that this is being compensated for in the cyanide deficient mutants by other mechanisms such as the accumulation and/or redirection of tyrosine into other metabolic pathways that may improve drought tolerance or to the synthesis and turnover of other specialised metabolites (Neilson et al. 2013; Hagerman et al. 1998).

Phenolics have been shown to possess antioxidant properties (Hagerman et al. 1998) and are often higher in plants experiencing drought stress, even cyanogenic plants, e.g. *Eucalyptus cladocalyx* (Gleadow and Woodrow 2002b). We measured the concentration of phenolics in various tissues of the *S. bicolor* plants to see if excess ROS generation was prevented in drought-stressed plants via biosynthesis of phenolic compounds rather than cyanogenic glucosides. We expected higher phenolics in the *tcd1* mutants than in the wildtype plants as compensation for lack of dhurrin. We did not observe any difference in the concentration of phenolics with treatment on a per mass or per plant basis, nor did we detect any correlation between the concentration of ROS and phenolics in either the wildtype or mutants lacking dhurrin.

## Conclusion

In this experiment, we compared the impact of drought on the growth and composition of *S. bicolor* genotypes that contain dhurrin with those that do not. We found no correlation between the concentration of ROS and the concentration of dhurrin or phenolics. Our results do not support the hypothesis that dhurrin synthesis and recycling are directly involved in mitigating ROS damage under the set of conditions imposed here. We propose that the well documented increase in dhurrin in droughted *S. bicolor* plants may function primarily in protecting leaves from any additional biotic stress incurred by herbivores that may occur in the field, or act as a store of nitrogen to promote post-drought recovery.

### *Author contribution statement*

Material preparation, data collection and analysis were performed by MNS, AAQ and CKB. The first draft of the manuscript was written by MNS. RMG conceived the project and extensively edited the manuscript. All authors commented on previous versions of the manuscript. All authors read and approved the final manuscript.

## Supplementary Information

Below is the link to the electronic supplementary material.

Supplementary file1 (DOCX 438 KB)

## Declarations

### Data availability

All data will be made available on request.
