# Supplementary material for: Dhurrin increases but does not mitigate oxidative stress in droughted Sorghum bicolor
Source: Planta. 2022 Feb 28;255(4):74. doi: 10.1007/s00425-022-03844-z (PMC8885504; doi:10.1007/s00425-022-03844-z)
Supplement: Supplementary file 1 — Supplementary file1 (DOCX 438 KB) [file 425_2022_3844_MOESM1_ESM.docx]

# Supplementary Information

**Dhurrin increases but does not mitigate oxidative stress in droughted *Sorghum bicolor***

M. N. Sohail^1,2^, A.A. Quinn^1^, C.K. Blomstedt^1^, and R.M. Gleadow^1^

^1^School of Biological Sciences, Monash University, Clayton, Vic. 3800, Australia.

^2^Present address; School of Natural Sciences, University of Tasmania, Private Bag 55, Hobart, Tasmania, 7001, Australia.

**Corresponding author**

^*^R.M. Gleadow; School of Biological Sciences, Monash University, Vic. 3800, Australia.

Telephone: +61 3 9905 1667

Email: ros.gleadow@monash.edu


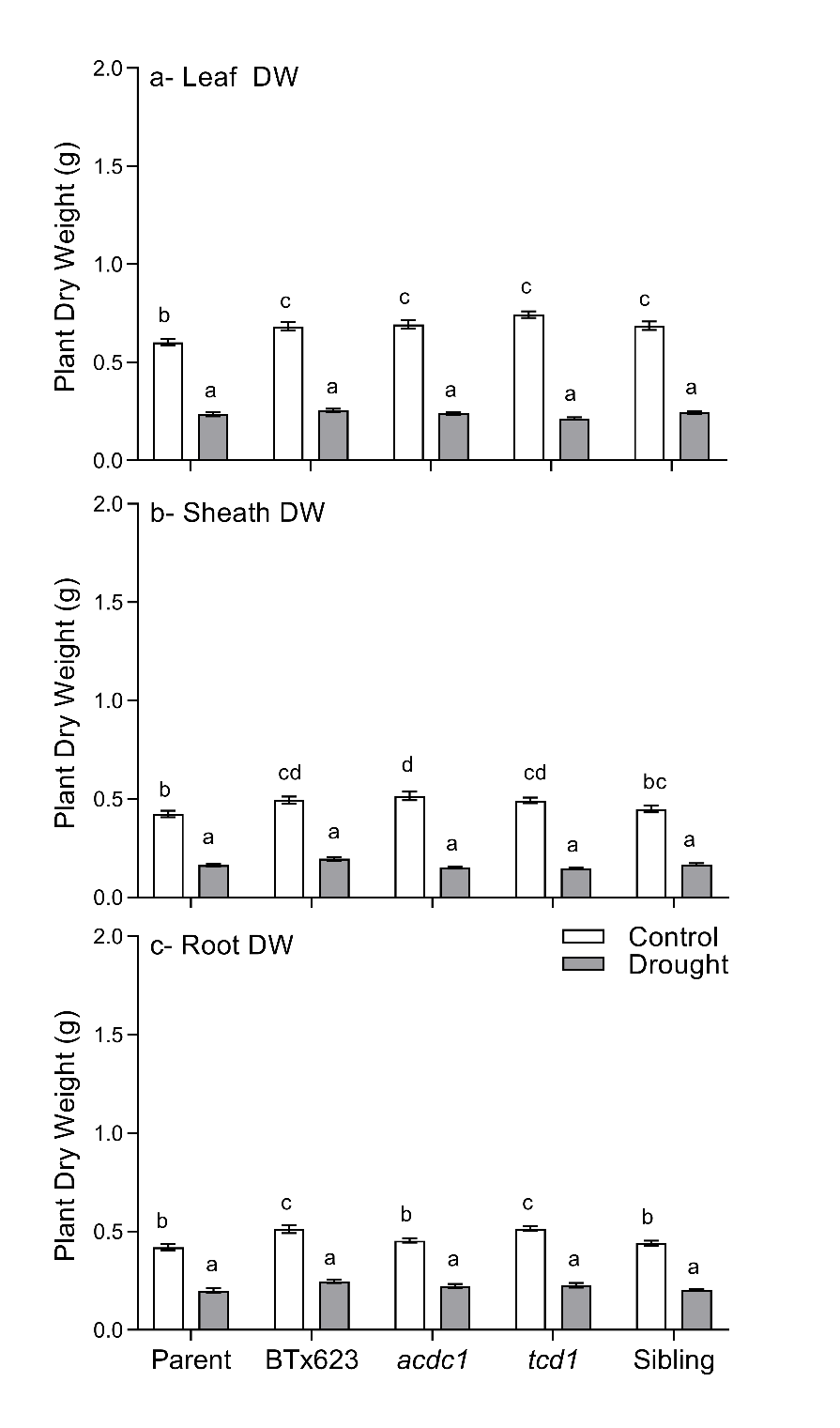


**Supplementary Figure S1:** Biomass (Dry weight, DW) of (a) leaves (leaf blades); (b) sheath (leaf sheath plus stems), and (c) roots of five different Sorghum genotypes (Parent, BTx623, *acdc1*, *tcd1* and sibling) grown under well-watered and drought conditions. *acdc1* plants had reduced levels of dhurrin compared to wildtype and *tcd1* plants lack dhurrin completely. The parental line is the one that was treated with EMS to create the mutants. Plants in the sibling line have the same genetic background as the mutants without the mutation and as included as a control. Plants (N=15) were harvest at the 5-6 leaf stage; a) total plant DW; b) leaf DW; c) plant sheath DW; d) root DW. Values are means ± SE. Columns with the same letter are not significantly different from other columns on the same graph (p>0.05).


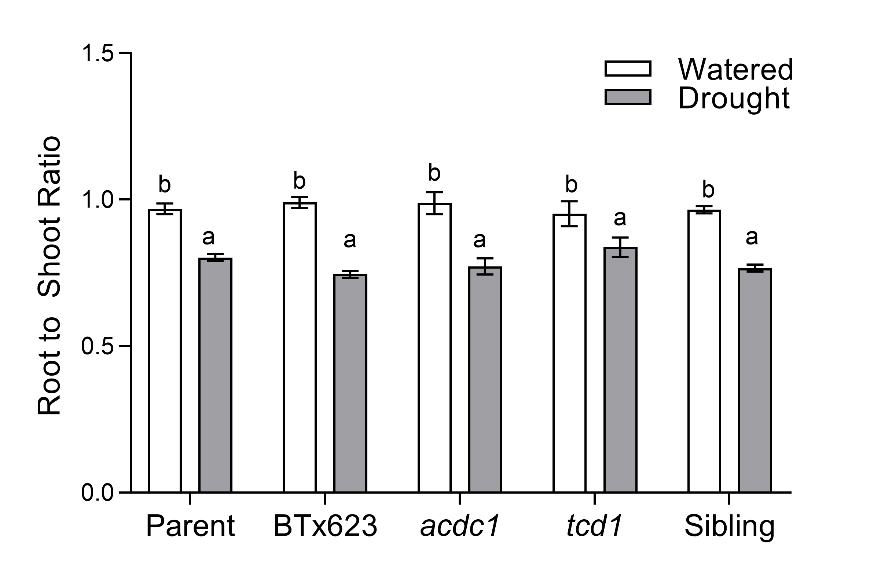


**Supplementary Figure S2.** Root to Shoot ratio of five different Sorghum genotypes (Parent, BTx623, *acdc1*, *tcd1* and sibling) grown under watered or droughted conditions for 2 weeks. Values are means of 15 replicated ± SE. Columns with the same letter are not significantly different from other columns on the same graph (p>0.05).


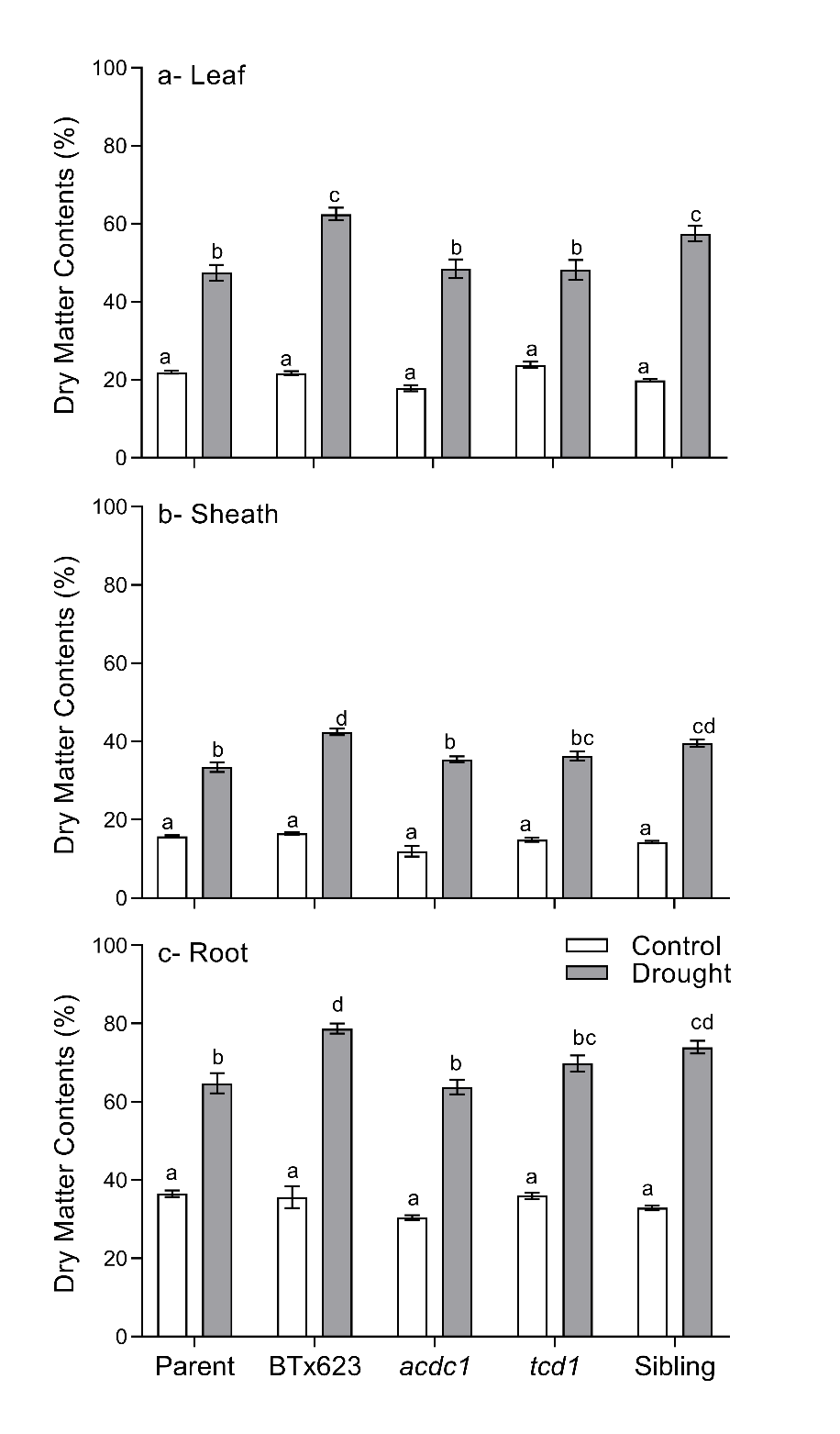


**Supplementary Figure S3**. Total Dry Matter content of (a) leaves (leaf blades); (b) sheath (leaf sheath plus stems), and (c) roots of five different Sorghum genotypes (Parent, BTx623, *acdc1*, *tcd1* and sibling) grown under well-watered or droughted conditions. Dry Matter Content (%) was calculated by dividing the dry mass for each tissue type by the fresh mass of the same tissue multiplied by 100. *acdc1* plants had reduced levels of dhurrin compared to wildtype and *tcd1* plants lack dhurrin completely. The parental line is the one that was treated with EMS to create the mutants. Plants in the sibling line have the same genetic background as the mutants without the mutation and as included as a control. Plants (N=15) were harvest at the 5-6 leaf stage; a) total plant DW; b) leaf DW; c) plant sheath DW; d) root DW. Values are means ± SE. Columns with the same letter are not significantly different from other columns on the same graph (p>0.05).
